# Supplementary material for: Effects of a Rice-Based Diet in Korean Adolescents Who Habitually Skip Breakfast: A Randomized, Parallel Group Clinical Trial
Source: Nutrients. 2021 Mar 5;13(3):853. doi: 10.3390/nu13030853 (PMC8002194; doi:10.3390/nu13030853)
Supplement: Supplementary file 1 [file nutrients-13-00853-s001.pdf]

Table S1. Clinical trial breakfast menu for the intervention and control groups (Examples)

Group	Mon.	Tue.	Wed.	Thur.	Fri.
Rice based meal	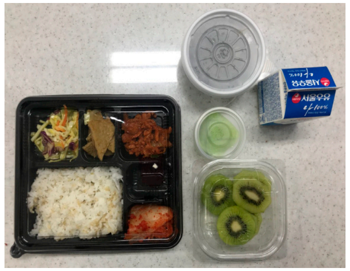	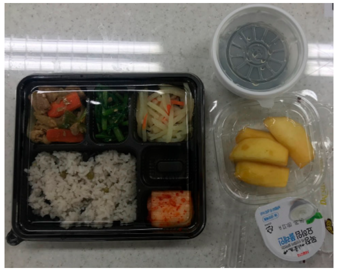	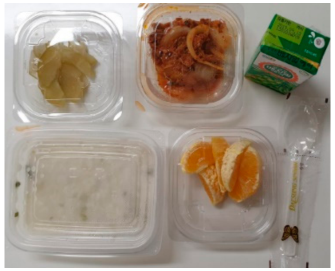	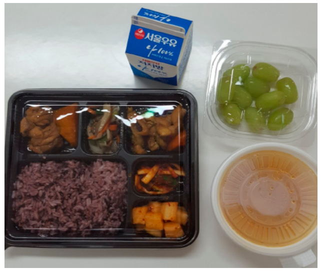	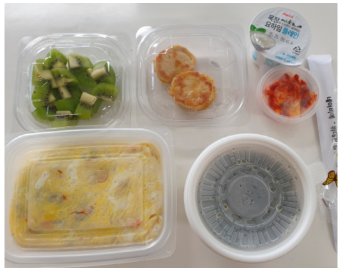
	Brown rice	Black bean rice	Chicken porridge	Black rice	Omurice
	Mallow doenjang soup	Clam seaweed soup	-	Pork Kimchi Stew	Miso soup
	Duck bulgogi	Pork bulgogi with soy sauce	-	Sweet pumpkin steamed chicken	-
	Fried fishball	Fried potato onion	-	Fried oyster mushroom	-
	Radish and vegetables with mustard sauce	Seasoned young summer radish	Seasoned pickled Uloe	Seasoned cucumber	Boiled shrimp meatballs
	Kimchi	Kimchi	Fried Kimchi with tuna	Radish kimchi	Kimchi
	Fruit	Fruit	Fruit	Fruit	Fruit
	Milk	Yogurt	Soy milk	milk	Yogurt

Wheat based meal	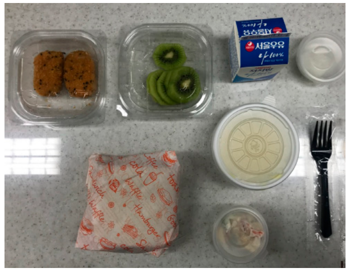	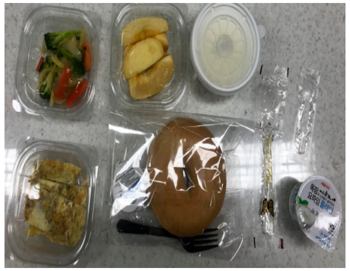	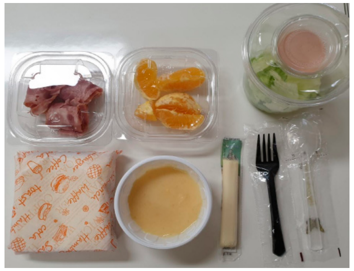	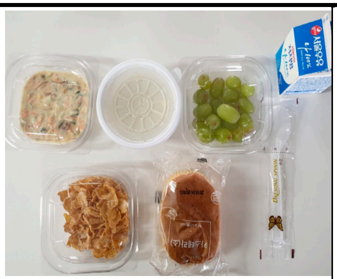	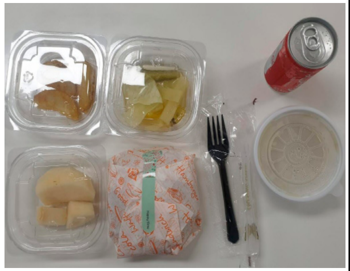
	Ham cheese sandwich	Bagels and cream cheese	French toast with cinnamon powder	Castella	Hamburger (chicken patty)
	Corn soup	Onion soup	Carrot soup	Broccoli soup	Mushroom soup
	Sweet potato croquette	Omelet	Bacon roast	Cereal	French fries with ketchup / Vegetable pickles
	Macaroni salad	Grilled vegetables	Vegetable salad	Sweet potato buckwheat	Carbonated drink
	Fruit	Fruit	Fruit	Fruit	Fruit
	Milk	Yogurt	String cheese	Milk	Cheese

Table 2. EEG analysis of the subjects*.

Variable	RMG (n=26)	WMG (n=29)	GMG (n=26)	*p*-value[1]	*p*-value[2]	*p*-value[3]	*p*-value[4]
			RT_F3				
0 wk	0.54±0.16	0.56±0.19	0.51±0.16				
12 wk	0.44±0.18	0.55±0.19	0.50±0.17		0.100	0.111	0.332
△ RT_F3	-0.10±0.21	-0.00±0.19	-0.01±0.19	0.130			
p-value[5]	0.019	0.956	0.729				
			RT_F4				
0 wk	0.54±0.18	0.54±0.18	0.50±0.17				
12 wk	0.44±0.19	0.54±0.19	0.48±0.20		0.204	0.178	0.670
△ RT_F4	-0.09±0.25	0.01±0.19	-0.02±0.22	0.235			
p-value[5]	0.073	0.841	0.664				
			RT_T3				
0 wk	056±0.17	0.59±0.22	0.56±0.18				
12 wk	0.47±0.20	0.57±0.19	0.58±0.20		0.195	0.235	0.535
△ RT_T3	-0.10±0.23	-0.02±0.26	0.02±0.25	0.256			
p-value[5]	0.040	0.698	0.761				
			RT_T4				
0 wk	0.53±0.19	0.56±0.21	0.50±0.19				
12 wk	0.43±0.19	0.54±0.20	0.48±0.21		0.395	0.130	0.568
△ RT_T4	-0.09±0.20	-0.02±0.24	-0.02±0.25	0.480			
p-value[5]	0.046	0.619	0.627				
			RT_P3				
0 wk	0.54±0.17	0.54±0.20	0.50±0.18				
12 wk	0.40±0.21	0.50±0.21	0.50±0.21		0.065	0.326	0.411
△ RT_P3	-0.14±0.22	-0.05±0.26	0.00±0.24	0.094			
p-value[5]	0.002	0.353	0.958				
			RT_P4				
0 wk	0.51±0.17	0.52±0.20	0.49±0.18				
12 wk	0.42±0.24	0.50±0.20	0.48±0.21		0.143	0.260	0.048
△ RT_P4	-0.12±0.24	-0.02±0.24	-0.00±0.23	0.189			
p-value[5]	0.020	0.656	0.947				

RT_O1							
0 wk	0.52±0.18	0.55±0.20	0.53±0.18				
12 wk	0.42±0.24	0.51±0.23	0.45±0.23		0.627	0.259	0.945
△ RT_O1	-0.10±0.23	-0.04±0.26	-0.07±0.33	0.703			
p-value[5]	0.037	0.448	0.258				
RT_O2							
0 wk	0.49±0.19	0.49±0.22	0.52±0.18				
12 wk	0.32±0.23	0.43±0.21	0.42±0.24		0.225	0.321	0.123
△ RT_O2	-0.18±0.30	-0.05±0.26	-0.07±0.26	0.266			
p-value[5]	0.007	0.265	0.063				
RA_F3							
0 wk	0.31±0.12	0.31±0.14	0.34±0.15				
12 wk	0.40±0.16	0.31±0.14	0.34±0.14		0.039	0.202	0.534
△ RA_F3	0.08±0.18	-0.00±0.13	0.00±0.13	0.062			
p-value[5]	0.024	0.953	0.962				
RA_F4							
0 wk	0.32±0.14	0.32±0.15	0.34±0.15				
12 wk	0.40±0.16	0.31±0.15	0.35±0.16		0.076	0.267	0.566
△ RA_F4	0.08±0.21	-0.01±0.13	0.01±0.17	0.104			
p-value[5]	0.061	0.648	0.710				
RA_T3							
0 wk	0.27±0.09	0.23±0.10	0.27±0.12				
12 wk	0.35±0.14	0.26±0.12	0.26±0.13		0.048	0.025	0.979
△ RA_T3	0.08±0.14	0.03±0.14	-0.01±0.14	0.061			
p-value[5]	0.006	0.254	0.634				
RA_T4							
0 wk	0.26±0.09	0.25±0.11	0.27±0.13				
12 wk	0.34±0.12	0.26±0.14	0.29±0.14		0.149	0.242	0.670
△ RA_T4	0.08±0.13	0.02±0.14	0.02±0.12	0.197			
p-value[5]	0.008	0.542	0.477				
RA_P3							
0 wk	0.33±0.15	0.33±0.17	0.36±0.17				
12 wk	0.45±0.19	0.36±0.18	0.37±0.18		0.045	0.347	0.476

△ RA_P3	0.12±0.19	0.03±0.19	0.01±0.18	0.079			
p-value[5]	0.003	0.401	0.851				
				RA_P4			
0 wk	0.35±0.14	0.34±0.17	0.37±0.17				
12 wk	0.45±0.19	0.36±0.17	0.37±0.18		0.057	0.335	0.074
△ RA_P4	0.13±0.21	0.02±0.17	-0.00±0.15	0.076			
p-value[5]	0.017	0.621	0.933				
				RA_O1			
0 wk	0.35±0.16	0.32±0.15	0.34±0.20				
12 wk	0.41±0.19	0.33±0.16	0.40±0.20		0.459	0.139	0.841
△ RA_O1	0.06±0.19	0.01±0.19	0.06±0.26	0.539			
p-value[5]	0.100	0.829	0.235				
				RA_O2			
0 wk	0.36±0.16	0.38±0.20	0.35±0.17				
12 wk	0.50±0.21	0.38±0.17	0.43±0.22		0.026	0.355	0.086
△ RA_O2	0.14±0.22^{Aa}	-0.00±0.19^{b}	0.08±0.22^{ab}	0.041			
p-value[5]	0.003	0.975	0.063				
				RB_F3			
0 wk	0.31±0.12	0.31±0.14	0.34±0.15				
12 wk	0.40±0.16	0.31±0.14	0.34±0.14		0.671	0.526	0.315
△ RB_F3	0.08±0.18	-0.00±0.13	0.00±0.13	0.062			
p-value[5]	0.024	0.953	0.962				
				RB_F4			
0 wk	0.32±0.14	0.32±0.15	0.34±0.15				
12 wk	0.40±0.16	0.31±0.15	0.35±0.16		0.854	0.793	0.860
△ RB_F4	0.08±0.21	-0.01±0.13	0.01±0.17	0.104			
p-value[5]	0.061	0.648	0.710				
				RB_T3			
0 wk	0.27±0.09	0.23±0.10	0.27±0.12				
12 wk	0.35±0.14	0.26±0.12	0.26±0.13		0.562	0.775	0.299
△ RB_T3	0.08±0.14	0.03±0.14	-0.01±0.14	0.061			
p-value[5]	0.006	0.254	0.634				
				RB_T4			

0 wk	0.26±0.09	0.25±0.11	0.27±0.13				
12 wk	0.34±0.12	0.26±0.14	0.29±0.14		0.708	0.486	0.834
△ RB_T4	0.08±0.13	0.02±0.14	0.02±0.12	0.197			
p-value[5]	0.008	0.542	0.477				
RB_P3							
0 wk	0.33±0.15	0.33±0.17	0.36±0.17				
12 wk	0.45±0.19	0.36±0.18	0.37±0.18		0.220	0.712	0.480
△ RB_P3	0.12±0.19	0.03±0.19	0.01±0.18	0.079			
p-value[5]	0.003	0.401	0.851				
RB_P4							
0 wk	0.35±0.14	0.34±0.17	0.37±0.17				
12 wk	0.45±0.19	0.36±0.17	0.37±0.18		0.705	0.669	0.126
△ RB_P4	0.13±0.21	0.02±0.17	-0.00±0.15	0.076			
p-value[5]	0.017	0.621	0.933				
RB_O1							
0 wk	0.35±0.16	0.32±0.15	0.34±0.20				
12 wk	0.41±0.19	0.33±0.16	0.40±0.20		0.581	0.859	0.949
△ RB_O1	0.06±0.19	0.01±0.19	0.06±0.26	0.548			
p-value[5]	0.100	0.829	0.235				
RB_O2							
0 wk	0.36±0.16	0.38±0.20	0.35±0.17				
12 wk	0.50±0.21	0.38±0.17	0.43±0.22		0.551	0.716	0.731
△ RB_O2	0.14±0.22[a]	-0.00±0.19[b]	0.08±0.22[ab]	0.041			
p-value[5]	0.003	0.975	0.063				
RSMT_F3							
0 wk	0.25±0.26	0.21±0.16	0.22±0.11				
12 wk	0.30±0.17	0.19±0.13	0.25±0.19		0.346	0.179	0.314
△ RSMT_F3	0.05±0.22	-0.01±0.15	0.04±0.19	0.371			
p-value[5]	0.227	0.592	0345				
RSMT_F4							
0 wk	0.26±0.30	0.22±0.16	0.23±0.14				
12 wk	0.29±0.17	0.21±0.16	0.30±0.30		0.603	0.319	0.781
△ RSMT_F4	0.03±0.25	-0.01±0.15	0.07±0.30	0.513			

p-value[5]	0.524	0.722	0.286				
RSMT_T3							
0 wk	0.25±0.24	0.32±0.52	0.23±0.15				
12 wk	0.35±0.25	0.22±0.18	0.23±0.21		0.175	0.334	0.318
△ RSMT_T3	0.10±0.27	-0.10±0.50	-0.00±0.24	0.134			
p-value[5]	0.072	0.285	0.945				
RSMT_T4							
0 wk	0.31±0.33	0.25±0.28	0.29±0.19				
12 wk	0.40±0.26	0.25±0.23	0.33±0.29		0.462	0.168	0.252
△ RSMT_T4	0.09±0.34	-0.01±0.28	0.05±0.32	0.549			
p-value[5]	0.216	0.893	0.458				
RSMT_P3							
0 wk	0.23±0.21	0.22±0.21	0.26±0.17				
12 wk	0.40±0.30	0.25±0.19	0.26±0.21		0.038	0.119	0.747
△ RSMT_P3	0.17±0.27^{a}	0.03±0.26^{b}	0.00±0.22^{b}	0.045			
p-value[5]	0.004	0.602	0.961				
RSMT_P4							
0 wk	0.24±0.14	0.26±0.26	0.24±0.14				
12 wk	0.41±0.31	0.24±0.19	0.28±0.24		0.016	0.098	0.396
△ RSMT_P4	0.17±0.27	-0.01±0.31	0.04±0.26	0.052			
p-value[5]	0.003	0.836	0.434				
RSMT_O1							
0 wk	0.26±0.31	0.23±0.21	0.23±0.19				
12 wk	0.46±0.44	0.25±0.24	0.39±0.50		0.387	0.249	0.611
△ RSMT_O1	0.20±0.43	0.06±0.32	0.15±0.45	0.438			
p-value[5]	0.026	0.299	0.091				
RSMT_O2							
0 wk	0.24±0.15	0.25±0.24	0.20±0.09				
12 wk	0.66±0.61	0.31±0.23	0.45±0.56		0.025	0.085	0.313
△ RSMT_O2	0.42±0.60^{a}	0.06±0.33^{b}	0.25±0.56^{ab}	0.034			
p-value[5]	0.001	0.345	0.032				

Data shown as mean±SD. F3: Left frontal lobe; F4: Right frontal lobe; GMG: general meal group; RA: relative alpha; RB: relative beta; RMG: rice meal group;

RSMT: ratio of SMR~mid beta to theta; RT: relative theta; SMR: sensory moto rhythm; T3: left temporal lobe; T4: right temporal lobe; O1: left occipital lobe; O2: right occipital lobe; P3: left parietal lobe; P4: right parietal lobe; wk week; WMG: wheat meal group.

△ = 12 week – 0 week. *EEG analysis of 81 study subjects with suitable meal compliance among per protocol set. [1]Analyzed by GLM (difference between change value, RMG vs. WMG vs. GMG) after adjusting for age, sex, school, physical activity; [2-4]Interaction, group effect, and time effect of two-way repeated measure ANOVA, respectively; [5]Analyzed by paired t-test between 0 week and 12 week.

Table S3. The nutrient intakes of the subjects*.

	**RMG (n=26)**	**WMG (n=29)**	**GMG (n=26)**	***P*-value[1]**	***P*-value[2]**	***P*-value[3]**	***P*-value[4]**
Energy (kcal/d)							
0 wk	1,784.97±596.38	1,661.45±585.18	1,781.19±420.57	0.349	0.815	0.226	0.440
12 wk	1,901.17±661.23	2,011.75±524.98	1,832.18±411.72	0.553	0.916	0.469	0.716
△Intake	116.20±681.44	350.30±539.15	50.99±52.51	0.168	0.697	0.086	0.171
P-value[5]	0.393	0.003	0.631				
Carbohydrate (g/d)							
0 wk	227.13±69.05	214.61±78.81	232.52±56.20	0.466	0.568	0.303	0.476
12 wk	251.97±85.59	264.48±76.12	243.25±57.17	0.598	0.788	0.571	0.730
△Intake	24.84±77.24	49.87±76.11	10.73±77.95	0.219	0.414	0.134	0.169
P-value[5]	0.114	0.003	0.498				
Protein (g/d)							
0 wk	65.64±22.62	61.10±24.61	65.51±19.81	0.338	0.912	0.222	0.445
12 wk	72.85±30.87	77.04±28.04	71.06±20.74	0.694	0.863	0.818	0.912
△Intake	7.21±32.88	15.93±34.88	5.55±21.08	0.370	0.964	0.263	0.482
P-value[5]	0.274	0.031	0.200				
Fat (g/d)							
0 wk	65.82±30.56	59.56±30.73	63.30±20.21	0.376	0.899	0.450	0.569
12 wk	64.31±30.93	69.57±22.07	62.33±17.55	0.541	0.860	0.402	0.741
△Intake	-1.51±37.07	10.01±34.48	-0.97±22.56	0.256	0.886	0.218	0.363
P-value[5]	0.837	0.160	0.832				
SFA (g/d)							

0 wk	7.70±3.74	9.40±6.20	10.41±6.00	0.269	0.023	0.548	0.167
12 wk	9.26±6.83	11.92±6.86	8.81±4.30	0.147	0.939	0.085	0.183
△Intake	1.57±6.82	2.52±9.93	-1.80±7.90	0.681	0.123	0.126	0.181
P-value[5]	0.253	0.216	0.312				
MUFA (g/d)							
0 wk	9.55±4.63	11.62±5.64	11.88±5.52	0.190	0.035	0.714	0.172
12 wk	12.15±8.71	15.08±8.47	10.89±4.98	0.191	0.916	0.060	0.177
△Intake	2.61±9.70	3.47±10.61	-1.14±7.22	0.726	0.143	0.100	0.190
P-value[5]	0.183	0.116	0.493				
PUFA (g/d)							
0 wk	10.65±5.72	10.23±4.21	10.17±4.74	0.705	0.747	0.737	0.937
12 wk	11.47±6.74	13.63±8.81	9.38±3.89	0.285	0.183	0.032	0.101
△Intake	0.83±9.16	3.40±9.00	-0.97±5.47	0.270	0.431	0.066	0.156
P-value[5]	0.648	0.071	0.486				
Fiber, g/d							
0 wk	14.49±4.31	12.55±4.01	14.67±5.39	0.084	0.685	0.153	0.146
12 wk	17.45±6.89	16.90±5.71	15.10±4.49	0.839	0.196	0.360	0.351
△Intake	2.95±7.81	4.34±5.57	0.27±6.84	0.371	0.172	0.053	0.086
P-value[5]	0.065	0.001	0.737				
Vitamin A (ug RAE/d)							
0 wk	317.48±140.60	336.24±107.92	367.37±166.86	0.503	0.198	0.607	0.416
12 wk	378.68±189.65	438.39±146.89	375.26±122.60	0.256	0.733	0.275	0.446
△Intake	61.20±243.29	102.14±143.99	2.62±200.11	0.602	0.462	0.175	0.426
P-value[5]	0.211	0.002	0.843				
Vitamin D, ug/d							
0 wk	1.94±1.63	2.34±1.79	1.70±1.04	0.481	0.737	0.293	0.542
12 wk	2.16±2.23	2.76±1.72	3.00±2.60	0.380	0.088	0.565	0.221
△Intake	0.22±2.55	0.42±2.41	1.33±2.62	0.847	0.081	0.253	0.215
P-value[5]	0.663	0.389	0.021				
Vitamin E (mg/d)							
0 wk	16.85±9.05	14.47±5.64	16.57±6.48	0.219	0.957	0.390	0.403
12 wk	17.83±9.12	17.53±5.47	14.21±4.52	0.928	0.146	0.034	0.173
△Intake	0.98±13.86	3.07±5.75	-2.36±7.72	0.441	0.278	0.015	0.136

P-value[5]	0.722	0.014	0.139				
Vitamin K (ug/d)							
0 wk	83.04±87.19	102.42±80.62	77.97±71.11	0.494	0.772	0.379	0.758
12 wk	85.07±50.25[b]	138.33±85.94[a]	86.86±60.87[b]	0.006	0.859	0.034	0.008
△Intake	2.03±102.32	35.91±112.42	9.19±74.59	0.179	0.904	0.352	0.283
P-value[5]	0.920	0.123	0.556				
Thiamine (mg/d)							
0 wk	1.62±0.51	1.68±0.69	2.10±2.17	0.847	0.181	0.161	0.214
12 wk	1.92±1.10	1.88±0.62	1.60±0.49	0.703	0.335	0.24	0.488
△Intake	0.31±1.13	0.19±0.81	-0.51±2.03	0.667	0.089	0.067	0.094
P-value[5]	0.181	0.255	0.231				
Rivoflavin (mg/d)							
0 wk	1.22±0.46	1.29±0.49	1.25±0.40	0.646	0.640	0.792	0.843
12 wk	1.40±0.54	1.54±0.39	1.37±0.41	0.264	0.793	0.230	0.490
△Intake	0.17±0.61	0.25±0.56	0.11±0.59	0.678	0.906	0.522	0.834
P-value[5]	0.165	0.034	0.333				
Vitamin C (mg/d)							
0 wk	42.73±33.38	42.12±26.15	46.81±25.87	0.945	0.577	0.855	0.852
12 wk	48.61±29.24	55.56±21.52	44.40±26.87	0.247	0.401	0.135	0.199
△Intake	5.88±47.04	13.43±34.26	-2.41±31.27	0.454	0.352	0.230	0.327
P-value[5]	0.530	0.062	0.704				
Folate (ug/d)							
0 wk	277.63±112.90	289.90±109.53	274.93±102.66	0.748	0.888	0.568	0.929
12 wk	331.86±128.74[ab]	382.04±105.78[a]	306.67±78.56[b]	0.082	0.449	0.013	0.041
△Intake	54.23±175.59	92.14±131.62	31.74±114.54	0.344	0.513	0.180	0.303
P-value[5]	0.128	0.002	0.179				
Vitamin B6 (mg/d)							
0 wk	1.59±1.20	1.44±0.75	1.43±0.61	0.438	0.736	0.841	0.723
12 wk	1.94±1.27	1.54±0.56	1.52±0.70	0.151	0.181	0.446	0.177
△Intake	0.34±1.84	0.10±0.65	0.09±0.78	0.623	0.463	0.697	0.690
P-value[5]	0.350	0.458	0.585				
Vitamin B12 (ug/d)							
0 wk	3.54±2.08 [b]	4.42±2.60 [ab]	5.90±3.02[a]	0.209	0.003	0.056	0.005

12 wk	5.18±2.80	5.52±4.46	5.99±2.52	0.689	0.293	0.438	0.458
△Intake	1.64±3.51	1.10±3.55	0.09±3.85	0.585	0.180	0.353	0.343
P-value[5]	0.025	0.134	0.907				
Biotin (ug/d)							
0 wk	0.47±0.79	1.14±1.32	1.21±1.32	0.019	0.033	0.880	0.057
12 wk	1.15±1.55 [b]	2.81±1.66 [a]	0.45±0.69 [b]	0.001	0.050	0.0001	0.0001
△Intake	0.68±1.53 [a]	1.67±2.50 [a]	-0.76±1.52 [B]	0.119	0.004	0.001	0.001
P-value[5]	0.033	0.003	0.020				
Calcium (mg/d)							
0 wk	322.53±163.09	339.40±148.10	373.40±154.21	0.551	0.229	0.623	0.510
12 wk	387.87±158.41	469.41±164.17	456.60±171.13	0.058	0.134	0.798	0.134
△Intake	65.34±159.39	130.01±168.16	78.72±199.66	0.225	0.773	0.478	0.773
P-value[5]	0.047	0.001	0.046				
Iron (mg/d)							
0 wk	10.14±4.38[b]	9.93±3.33[b]	15.26±10.69[a]	0.775	0.019	0.011	0.004
12 wk	13.90±8.56	13.48±4.38	13.44±5.59	0.899	0.660	0.887	0.973
△Intake	3.76±7.96	3.55±4.29	-1.90±11.13	0.973	0.045	0.024	0.504
P-value[5]	0.024	0.0001	0.422				
Magnesium (mg/d)							
0 wk	64.69±30.06	67.68±29.56	80.18±40.92	0.727	0.039	0.177	0.407
12 wk	82.76±38.41	90.19±36.28	97.29±32.21	0.358	0.099	0.354	0.401
△Intake	18.07±50.71	22.51±39.30	15.97±44.85	0.603	0.514	0.476	0.574
P-value[5]	0.081	0.009	0.021				

Data shown as Mean±SD. GMG: general meal group; RMG: rice meal group; SFA: saturated fatty acid; wk: week; WMG: wheat meal group; PUFA: polyunsaturated fatty acid. △intake = 12 wk - 0wk. * the nutrient intake of 81 study subjects with suitable meal compliance among per protocol set.
[1]Analyzed by ANCOVA between RMG group and WMG group after adjusting for age, sex and school. [2]Analyzed by ANCOVA between RMG group and GMG group after adjusting for age, sex and school. [3]Analyzed by ANCOVA between WMG group and GMG group after adjusting for age, se and, school. [4]Analyzed by ANCOVA among three groups (RMG vs. WMG vs. GMG) after adjusting for age, sex, and school. [5]Analyzed by paired t-test between 0 week and 12 week.
